# Prognostic Stratification of Metastatic Prostate Cancer Patients Treated With Abiraterone and Enzalutamide Through an Integrated Analysis of Circulating Free microRNAs and Clinical Parameters

**DOI:** 10.3389/fonc.2021.626104

**Published:** 2021-03-16

**Authors:** Evgeniya Sharova, Marco Maruzzo, Paola Del Bianco, Ilaria Cavallari, Francesco Pierantoni, Umberto Basso, Vincenzo Ciminale, Vittorina Zagonel

**Affiliations:** ^1^Immunology and Molecular Oncology Unit, Veneto Institute of Oncology IOV – IRCCS, Padua, Italy; ^2^Oncology 1 Unit, Department of Oncology, Veneto Institute of Oncology IOV – IRCCS, Padua, Italy; ^3^Clinical Research Unit, Veneto Institute of Oncology IOV – IRCCS, Padua, Italy; ^4^Department of Surgery, Oncology and Gastroenterology, University of Padua, Padua, Italy

**Keywords:** mCRPC, cfmiRNA, abiraterone, enzalutamide, OS, PFS, prognostic biomarkers

## Abstract

Androgen Receptor-Targeted Agents (ARTA) have dramatically changed the therapeutic landscape of metastatic Castration-Resistant Prostate Cancer (mCRPC), but 20–40% of these patients progress early after start of ARTA treatment. The present study investigated the potential utility of plasma cell-free microRNAs (cfmiRNAs) as prognostic markers by analyzing a prospective cohort of 31 mCRCP patients treated with abiraterone (*N* = 10) or enzalutamide (*N* = 21). Additional potential prognostic factors were extracted from clinical records and outcome was evaluated as overall survival (OS) and progression-free survival (PFS). cfmiRNAs were measured in plasma samples using quantitative real-time RT-PCR. Linear correlation among clinical factors and cfmiRNAs was assessed using the Spearman's rank correlation coefficient. The association with survival was studied using univariate and multivariate Cox proportional hazards models. Continuous variables were dichotomized with the cut points corresponding to the most significant relation with the outcome. Univariate analysis indicated that plasma levels of miR-21-5p, miR-141-3p and miR-223-3p, time to development of castration-resistance (tCRPC), and blood hemoglobin (Hb) levels strongly correlated with both PFS and OS. Multivariate analysis revealed that low plasma levels of miR-21, shorter tCRPC, and lower Hb values were independent factors predicting reduced PFS and OS. These findings suggest that the integrated analysis of cfmiRNAs, tCRPC, and Hb may provide a promising, non-invasive tool for the prognostic stratification of mCRPC patients treated with ARTA.

## Introduction

The fact that tumor cells from metastatic castration-resistant prostate cancer (mCRPC) patients are still somewhat addicted to androgen signaling (1) posed the rational base for the design of next-generation Androgen Receptor-Targeted Agents (ARTA) that achieve profound inhibition of androgen signaling in these patients. These compounds include abiraterone, a CYP17A1 inhibitor that blocks the synthesis of androgenic precursors, and enzalutamide, which antagonizes AR activation and nuclear translocation (2). The introduction of ARTA has considerably improved the overall survival (OS) of mCRPC patients from 12–18 months to approximately 3 years in docetaxel-naïve patients (3–5). However, the evaluation of response to ARTA is challenging. A decline in the levels of Prostate Specific Antigen (PSA) blood levels in the first 4 weeks of treatment with ARTA was demonstrated to be correlated with OS in large retrospective trials (6). However, a reduction in PSA cannot be considered as a predictive factor “*per se*” in all cases since paradoxical PSA surges have been described in patients treated with ARTA (7). Therefore, there is a pressing need for additional biomarkers for the early identification of relapse and to guide the choice of the best treatment for the individual patient.

Several studies have highlighted the role of microRNAs (miRNAs) in the pathogenesis of PCa (8); among these, miR-21 and miR-141 play key regulatory roles in activation of the epithelial-mesenchymal transition (EMT) program, and their expression in cancer cells is correlated with patients' prognosis and response to therapy (8, 9). Circulating free miRNAs (cfmiRNAs) released by cancer cells as well as by cells of the tumor microenvironment are emerging as promising markers of disease, as they are resistant to degradation and are readily quantifiable. Our pilot study was aimed at investigating the possible relationship between cfmiRNAs and the clinical outcome of mCRPC patients treated with ARTA.

## Materials and Methods

### Study Design and Patients

This exploratory prospective observational study was performed on a cohort of 31 mCRPC patients treated with abiraterone (10 patients) or enzalutamide (21 patients); ARTA was administered either as first-line therapy (26 patients) or after treatment with docetaxel (five patients). All consecutive patients who were candidates to receive ARTA and were eligible according to the study criteria were enrolled between September 2016 and October 2017 at the Veneto Institute of Oncology. The study was conducted according to the Declaration of Helsinki and approved by the local Ethics Committee; all patients signed an informed consent form prior to their inclusion.

Patients were selected according to the following inclusion criteria: (i) histological diagnosis of prostate cancer; (ii) metastatic disease at any site; (iii) mCRPC according to the Prostate Cancer Working Group 3 (PCWG3) definition (10); (iv) at least 6 months of life expectancy; (v) patients receiving bisphosphonates or antiresorptive drugs were included in the study if these treatments started before enrolment or after the first disease assessment. Patients with known cerebral lesions or impending spinal cord compression were excluded from study, as well as subjects with severe cardiovascular or metabolic diseases or swallowing problems contraindicating the administration of ARTA. Patients with previous exposure to second-line chemotherapy (cabazitaxel) or other second-line treatment were also excluded from the study.

At the start of ARTA treatment blood samples were collected for the miRNA analyses. All the patients were then treated as per clinical practice, according to the drugs' current label authorization and international guidelines for the treatment of mCRPC. ARTA therapy was administered until progression and clinical need to start another therapy, or when the patient experienced unacceptable toxicity or decided to withdraw from treatment. Disease progression was defined according to the PCWG3 criteria. No change in patients' management was introduced based on the results of the biomarker analysis. Comorbidities and contraindications to steroids guided the choice between abiraterone and enzalutamide. Adverse events were documented and treated in line with the best clinical practice.

Clinical examination and assessment of hematological and biochemical parameters were performed on a monthly basis during treatment. Disease restaging was performed every 3 to 4 months with serum PSA quantification and contrast-enhanced CT scan of the thorax, abdomen and pelvis plus bone scan, or with a total body CT/PET scan with ^18^F-choline. Plasma samples for miRNA analysis were obtained within 1 day before the start of ARTA. After disease progression, all the patients were followed up for survival, and received further lines of therapy or only best supportive care (BSC) according to their performance status and fitness to treatment, as indicated by the national and international guidelines for mCRPC.

### Sample Processing and miRNA Quantification

Blood samples were collected in EDTA-containing tubes at room temperature and processed for plasma isolation within 2 h as described by Cavallari et al. (11). Plasma samples were assayed for haemolysis (the presence of free hemoglobin corrected for lipoproteins) by measuring absorbance at 385 nm and 414 nm with a NanoDrop® ND-1000 UV-Vis spectrophotometer (Thermo Fisher Scientific) as described elsewhere (12) (Supplementary Table 1). Plasma samples were aliquoted and stored at −80°C. Total RNA (<1,000 nt/bp size range) was extracted from 300 μl of plasma with the Nucleo Spin miRNA plasma kit (Macherey-Nagel) following the manufacturer's instructions and eluted in 30 μl of RNAse-free water. Samples were analyzed for miRNA expression using specific TaqMan stem-loop reverse-transcription and PCR primer/probe assays (Thermo Fisher Scientific) in a Roche Light Cycler 480 thermal cycler as described by Sharova et al. (13). The following miRNAs were examined: hsa-miR-141-3p (Assay ID 000463), hsa-miR-223-3p (Assay ID 002295), and hsa-miR-21-5p (Assay ID 000397), chosen because of their reported relevance to mCRPC (14). Ct values obtained for the miRNAs of interest were normalized against the Ct values measured for hsa-miR-1228-3p (Assay ID 002919) using the formula 2^−ΔCt^= 2^−(*CtmiRx*−*CtmiR*1228)^. miR-1228 was chosen as the normalizer based on its prior use as a normalizer in studies of prostate cancer patients (15, 16). Our assays confirmed the low variability of miR-1228 levels in the plasma samples studied here (Supplementary Table 1).

### Statistical Analysis

Clinical variables to be tested as prognostic factors were PSA, type of ARTA, performance status, time to CRPC, neutrophil/lymphocyte ratio, hemoglobin and Gleason score. Quantitative variables were described as median and interquartile range, categorical variables were summarized as counts and percentages. The median follow-up time was based on the reverse Kaplan-Meier estimator. The association of patients' characteristics with the treatment received was assessed using the χ2 or Fisher exact test as appropriate. The linear correlation between continuous clinical variables and cfmiRNAs was assessed using the Spearman's rank correlation coefficient.

OS was defined as the time from the start of treatment with ARTA to death, and progression-free survival (PFS) was calculated from the start of treatment with ARTA to the date of radiological/clinical disease progression, or death. Patients who did not develop an event during the study period were censored at the date of the last observation. The cfmiRNAs were dichotomized with cut points corresponding to the most significant relation with the outcome, estimated from maximally selected log-rank statistic for values between the 10 and 90% quantiles using the upper bound of the *p*-value by Hothorn and Lausen (17).

Survival curves were estimated with the non-parametric Kaplan-Meier method and comparisons among strata were performed using the log-rank test. The 95% confidence interval (CI) for the median survival was calculated according to Brookmeyer and Crowley. Hazard ratios (HR) and 95% CI for each group were estimated using univariate Cox proportional hazards models with Efron's method of tie handling. No deviation from the proportional hazards assumption was found by the test statistic of Grambsch and Therneau (18). To assess the False-Discovery-Rate, *p*-values were adjusted by applying the Benjamini-Hochberg correction (19).

The independent role of each covariate in predicting survival was verified in a multivariable model considering all characteristics significantly associated with the outcome in the univariate analyses. All statistical tests were two-sided and a *p*-value <0.05 was considered statistically significant. Statistical analyses were performed using RStudio (RStudio: Integrated Development for R. RStudio Inc., Boston, MA, U.S.A.).

## Results

### Characteristics of the Patient Cohort

Thirty-one mCRPC patients were enrolled in the present study, 10 of whom were treated with abiraterone acetate and 21 with enzalutamide (Table 1). The median age was 75 years (range 69.5–80.5). Thirteen patients had stage IV disease at diagnosis of prostate cancer. All patients had been treated with ADT using Luteinizing Hormone Releasing Hormone (LHRH) analogs or antagonists after evidence of metastatic disease. The median time from start of ADT to castration resistance was 38.1 months. Five patients were previously exposed to docetaxel. Docetaxel-treated patients did not show a significant difference in the levels of the cfmiRNAs examined (miR-21, miR-141 and miR-223) compared to chemonaïve patients. 75% of all patients had a Gleason score greater than 8 at diagnosis. At the study's conclusion, 26 patients had progressed and 13 died. The median PFS was 19.3 months (95%CI 11.7–29.6) (Supplementary Table 2). The median OS was not reached (Supplementary Table 3). The median follow-up time was 36.6 months (95%CI: 35.4–39.3). Upon development of disease progression, 14 patients received only BSC, 10 were treated with docetaxel, 2 with cabazitaxel, 3 with Radium^223^, 1 with a second ARTA (abiraterone) and 1 with oral cyclophosphamide.

**Table 1:** Patients' characteristics before the start of ARTA.

		**Abiraterone Acetate (*N*=10)**	**Enzalutamide (*N*=21)**	**Total (*N* = 31)**	***P-*value**
Age, years	Median (Q1–Q3)	69.5 (66.5;73.2)	78 (73;82)	75 (69.5;80.5)	*0.031‡*
PSA, ng/ml	Median (Q1–Q3)	33.3 (8.2;65.7)	19.2 (9.4;53.4)	19.2 (8.4;58.6)	*0.767‡*
Time to CRCP, months	Median (Q1–Q3)	36.5 (19.5;48.9)	38.1 (19.2;56.4)	38.1 (19.1;53.1)	*0.800‡*
*N*, 10^9^/L	Median (Q1-Q3)	4.1 (2.7;5.1)	3.7 (2.7;5.0)	3.7 (2.7;5.0)	*0.767‡*
Ly, 10^9^/L	Median (Q1-Q3)	1.6 (1.1;2.1)	1.8 (1.4;2.4)	1.7 (1.3;2.3)	*0.228‡*
N/L	Median (Q1–Q3)	2.3 (1.4;4.3)	1.7 (1.4;2.4)	1.9 (1.4;3.0)	*0.375‡*
Hb, g/L	Median (Q1–Q3)	134.5 (129.2;135.0)	131.0 (122.0;135.0)	132.0 (124.5;135.0)	*0.421‡*
Site of metastasis	Lymph node	2 (20.0%)	5 (23.8%)	7 (22.6%)	*1.00°*
	Bone	5 (50.0%)	9 (42.8%)	14 (45.2%)	
	Lymph node, bone	3 (30.0%)	6 (28.6%)	9 (29.0%)	
	Visceral	0	1 (4.8%)	1 (3.2%)	
ECOG PS	0	6 (60.0%)	4 (19.0%)	10 (32.3%)	*0.040°*
	1–2	4 (40.0%)	17 (81.0%)	21 (67.7%)	
Gleason score	< =7 (5–7)	3 (30.0%)	4 (22.2%)	7 (25.0%)	*0.674°*
	>7 (8–10)	7 (70.0%)	14 (77.8%)	21 (75.0%)	
	Missing		3	3	

PSA, prostate-specific antigen; Time to CRPC, time to development of castration-resistance; N, neutrophils; Ly, lymphocytes; N/L, neutrophil-lymphocyte ratio; HB, hemoglobin; ECOG PS, Eastern Cooperative Oncology Group Performance Status. Data are expressed as median (interquartile range) for continuous data and n (percentage) for categorical data.

‡ *Kruskal-Wallis test*;

° *Fisher's Exact Test*.

### Plasma Levels of miR-21 and tCRPC Predict PFS of mCRPC Patients

A series of statistical analyses was performed to interrogate the possible prognostic value of plasma levels of miR-21, miR-141, and miR-223 (all normalized against miR-1228) measured for mCRPC patients at the start of ARTA treatment (see section Materials and Methods).

**Figure 1:**
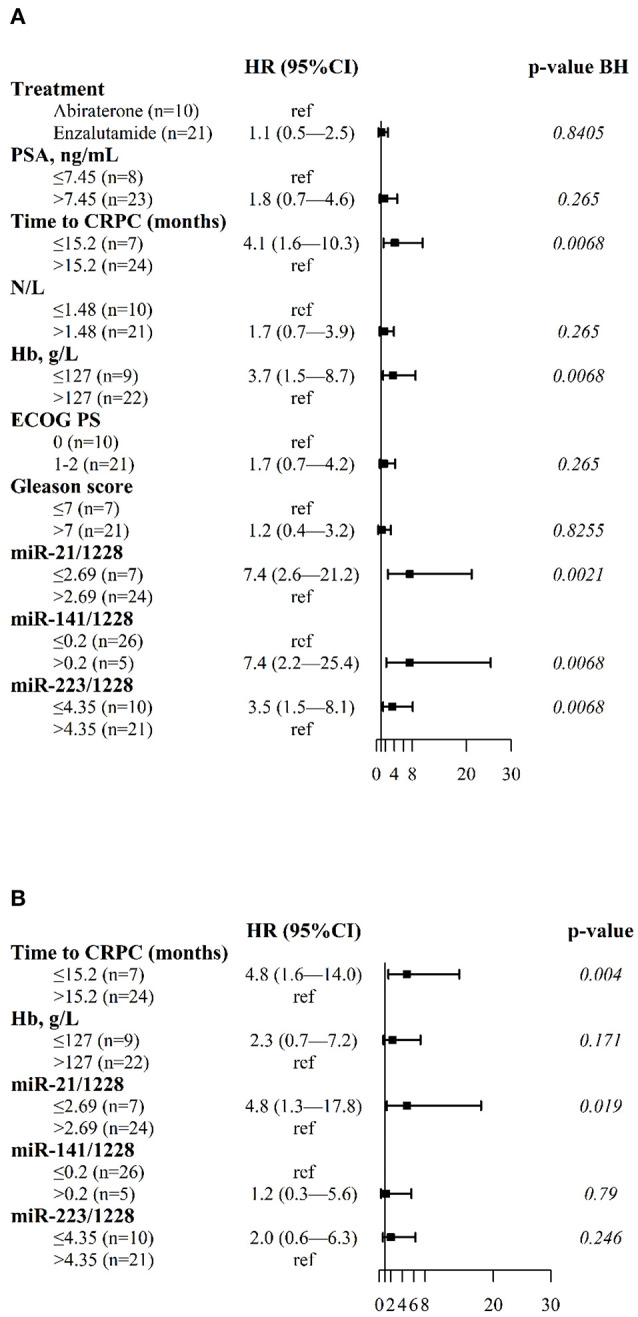
Forest plots of clinical variables and cfmiRNAs associated with PFS. **(A)** Univariate Cox regression model; **(B)** Multivariable Cox regression model. Indicated are the hazard ratios (HR) and 95% confidence intervals (CI) and corresponding *p*-values; *p*-value BH indicates the *p*-values determined using the Benjamini-Hochberg correction for multiple tests. Ref indicates the reference value for calculating the hazard ratio.

Univariate analysis indicated a significant association between plasma levels of miR-21, miR-141, miR-223 and PFS (Figure 1A and Supplementary Figure 1). Low plasma levels of miR-21 (2^−Δ*Ct*^ ≤2.69) and miR-223 (2^−Δ*Ct*^ ≤4.35) and high plasma miR-141 values (2^−Δ*Ct*^>0.20) were significantly associated with shorter PFS in all patients. In addition, shorter tCRPC (≤15.2 months) and low blood hemoglobin (≤127 g/L) were also significantly correlated with reduced PFS. N/L, PSA values, and the Gleason score at the start of ARTA treatment were not related to clinical outcome in our cohort.

Figure 1B shows results of multivariable Cox regression analysis, indicating that only normalized plasma miR-21 levels and tCRPC were independent predictors of PFS.

The median PFS was 2.1 months for patients with shorter tCRPC and low miR-21 values (95%CI: 2.1-NE), 11.7 months for patients with one risk factor (shorter tCRPC or low miR-21 values) (95%CI: 8.7-NE) and 29.3 months for patients with longer tCRPC and high miR-21 values (95%CI: 20.1_NE) (**Figure 3A**).

### Plasma Levels of miR-21, Anemia and tCRPC Are Independent Predictors of OS for mCRPC Patients

**Figure 2:**
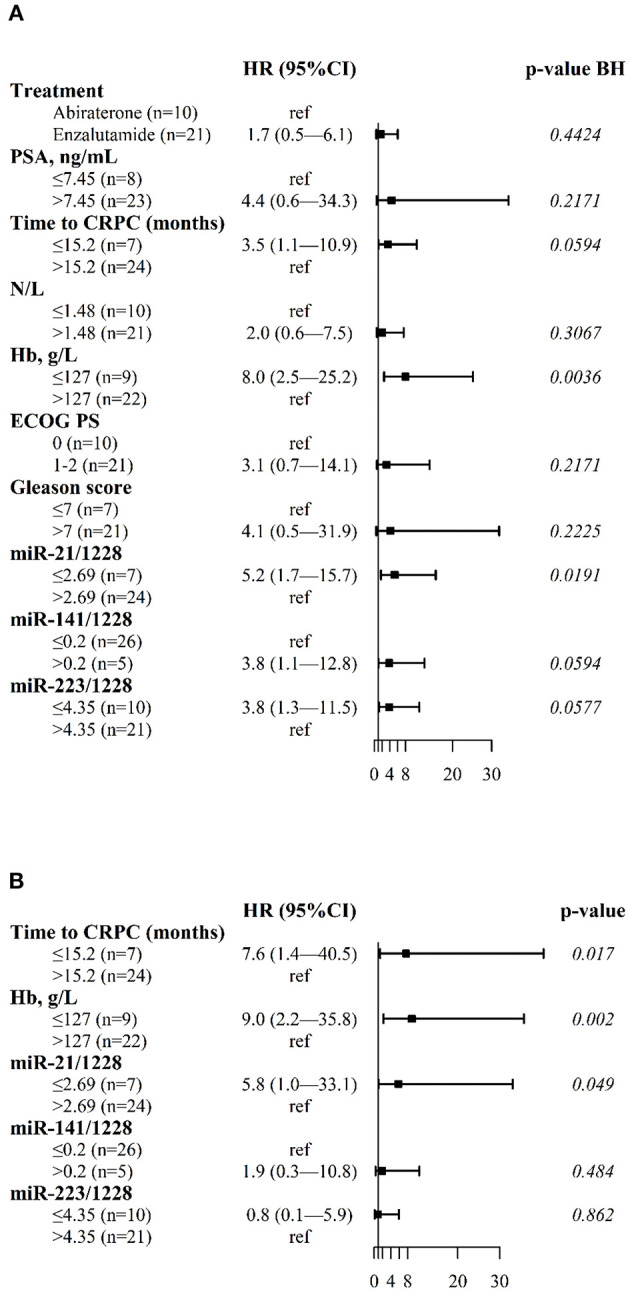
Forest plots of clinical variables and cfmiRNAs associated with OS. **(A)** Univariate Cox regression model **(B)** Multivariable Cox regression model. Indicated are the hazard ratios (HR) and 95% confidence intervals (CI) and corresponding *p*-values; *p*-value BH indicates the *p*-values determined using the Benjamini-Hochberg correction for multiple tests. Ref indicates the reference value for calculating the hazard ratio.

In univariate analysis, OS was correlated with the plasma levels of miR-21, miR-141, and miR-223, and with tCRPC and Hb (Figure 2A). Figure 2B shows the results of the multivariate Cox regression model revealing three independent factors predicting OS: plasma levels of miR-21, blood Hb and tCRPC.

**Figure 3:**
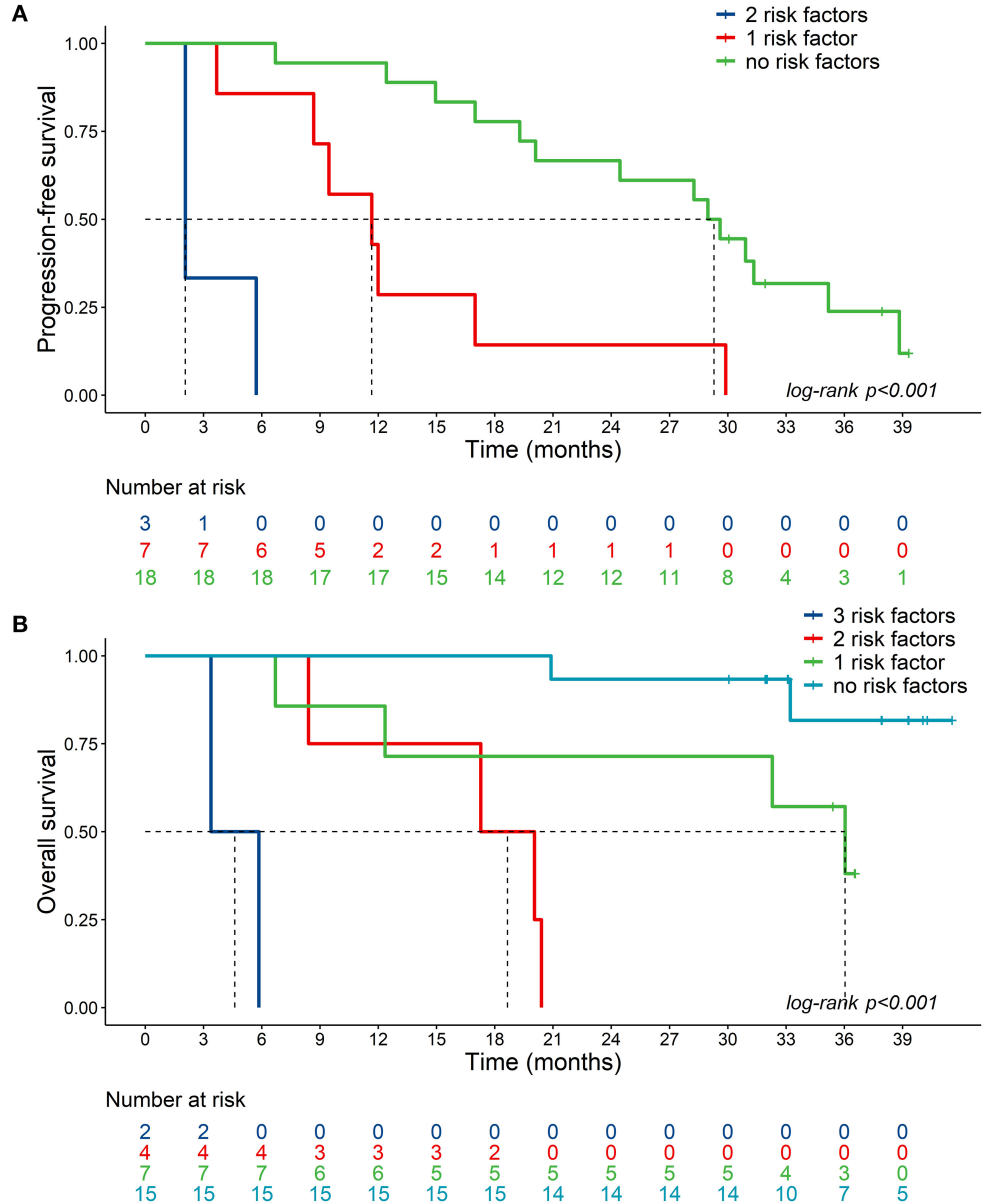
Kaplan-Meier plots. **(A)** Kaplan–Meier plots of PFS in mCRPC patients with high plasma miR-21 levels and long tCRPC (no risk factors, miR-21>2.69, tCRPC>15.2), low plasma miR-21 or short tCRPC (1 risk factor, miR-21≤2.69, tCRPC>15.2, or miR-21>2.69, tCRPC≤15.2), low plasma miR-21 and short tCRPC (2 risk factors, miR-21≤2.69, tCRPC≤15.2); **(B)** Kaplan–Meier plots of OS in mCRPC patients with high miR-21/1228, high hemoglobin and long tCRPC (no risk factors, miR-21>2.69, Hb>127, tCRPC>15.2); low plasma miR-21, or low hemoglobin or short tCRPC (1 risk factor, miR-21≤2.69, Hb>127, tCRPC>15.2 or miR-21>2.69, Hb≤127, tCRPC>15.2 or miR-21>2.69, Hb>127, tCRPC≤15.2); 2 out of 3 of these risk factors (miR-21≤2.69, Hb≤127, tCRPC>15.2 or miR-21≤2.69, Hb>127, tCRPC≤15.2 or miR-21>2.69, Hb≤127, tCRPC≤15.2); low plasma miR-21, low hemoglobin and short tCRPC (3 risk factors, miR-21≤2.69, Hb≤127, tCRPC≤15.2).

The median OS was not reached for patients with longer tCRPC, high hemoglobin concentration and high plasma miR-21 values (i.e., no risk factors). The median OS was 36.0 months (95%CI: 12.3-NE) for patients with one risk factor, 18.7 months (95%CI: 8.4-NE) for patients with two risk factors, and 4.6 months (95%CI: 3.4-NE) for patients with shorter tCRPC, low Hb concentration and low plasma miR-21 values (Figure 3B).

No significant correlation was found between plasma miR-21 levels and clinical variables (Supplementary Table 4). Plasma miR-21 levels were strongly correlated with plasma miR-223 levels (*r* = 0.71; *p* < 0.001) (Supplementary Table 4).

## Discussion

The present study explored the relationship between circulating miR-21, miR-141 and miR-223 and clinical outcome of patients with mCRPC treated with ARTA. Among the miRNAs tested, high expression of miR-21 was the best predictor of favorable PFS and OS after treatment with abiraterone or enzalutamide in our cohort of patients.

To date, few studies have analyzed circulating miR-21 in mCRPC. In an analysis of plasma/serum samples from 97 mCRPC patients, Lin et al. (20) found that low levels of miR-21 correlated with a shorter OS following treatment with docetaxel. A recent analysis by Benoist et al. of miRNAs in whole blood samples did not reveal significant differences in the levels of miR-21 or miR-141 in mCRPC patients treated with enzalutamide compared to healthy volunteers (21). However, miRNAs detected in whole blood samples will be predominantly represented by those contained in erythrocytes, platelets and leukocytes, which likely mask the contribution of circulating cell-free miRNAs.

The relationship between miR-21 and androgen-responsive signaling pathways was investigated by Mishra et al. who proposed a positive feedback loop mechanism in prostate cancer cells (22). Androgen receptor (AR) signaling directly enhances miR-21 gene transcription through androgen response elements (AREs) within the miR-21 promoter region (23). miR-21 in turn inhibits expression of the tumor suppressor protein phosphate and tensin homolog deleted on chromosome 10 (PTEN) (24), which negatively controls the AR. Interestingly, PTEN is frequently mutated or deleted in CRPC (25). These feedback connections between miR-21, the AR, and PTEN suggest that high plasma levels of miR-21 might be an indicator of increased AR activity in cancer cells, which is predictive of response to ARTA. This hypothesis is consistent with our results indicating that ARTA-treated patients with high plasma miR-21 levels might have a more favorable PFS and OS compared to those with low levels.

Our finding of higher plasma levels of miR-141 in patients with shorter PFS and OS in the univariate analysis is in line with the results of previous studies showing increased plasma/serum levels of miR-141 in patients with mCRPC compared to healthy/benign prostatic hyperplasia (BPH) subjects (26, 27) or patients with localized prostate cancer (28, 29). A member of the miR-200 family, miR-141 is involved in the epithelial-mesenchymal transition and is thus likely to play an important role in the clinical progression of PC (30). In addition, miR-141 was shown to target the expression of small heterodimer partner (SHP), a corepressor that blunts the activation of target genes by the AR (31). Our findings are coherent with the results of a recent study showing that high plasma levels of miR-141 are associated with shorter PFS/OS in a cohort of mCRPC patients (32).

We previously reported downregulation of cfmiR-223 in plasma from patients with localized PC compared to BPH controls (13), which is consistent with another study that revealed downregulation of miR-223 in CRPC and PC tissues compared to non-PC control samples (33). Consistent with these observations, Kurozumi et al. suggested a tumor suppressor role for this miRNA through regulation of the integrin receptors ITGA3 and ITGB1, a function that might affect the metastatic potential of PC cells (34). There is also evidence that miR-223 is abundantly expressed in macrophages and is transferred to malignant cells, where it inhibits their proliferation (35). Our finding that patients with low PFS/OS exhibit lower plasma levels of miR-223 may thus reflect a reduced immune response in patients with worse clinical outcome.

Multivariable analysis showed that the plasma level of miR-21 (but not miR-223 or miR-141) was associated with outcome (OS and PFS), independently of other clinical variables. In our evaluation of different clinical parameters usually included in prognostic/predictive models of metastatic prostate cancer (36) time to development of castration resistance and Hb levels were confirmed as independent prognostic factors for OS, findings in line with the results of previous studies of mCRPC patients treated with enzalutamide (37). Interestingly, low Hb levels are also associated with poor survival in several other tumor types (38).

The results presented here are promising, but must be considered preliminary, due to the limited number of patients examined, which reflects the exploratory nature of the study that implies the lack of any previous information required to carry out a power analysis and determine the appropriate sample size. In addition, our cohort included five patients who were previously treated with docetaxel. Although it is possible that docetaxel may alter tumor biology and influence miRNA expression, in the context of our study, docetaxel-treated patients did not show a significant difference in the levels of the cfmiRNA examined compared to chemonaïve patients; furthermore chemotherapy is currently administered at very early stages of disease and is not considered cross-resistant with ARTA.

Taken together, our findings suggest that the integration of the analysis of cfmiR-21 with the clinical parameters tCRPC and Hb levels may provide useful information for the prognostic stratification of mCRCP patients receiving ARTA treatment, and lay the ground for a large prospective validation study.

## Data Availability Statement

The original contributions presented in the study are included in the article/Supplementary Materials, further inquiries can be directed to the corresponding author/s.

## Ethics Statement

The studies involving human participants were reviewed and approved by Ethics Committee of the Veneto Institute of Oncology. The patients/participants provided their written informed consent to participate in this study.

## Author Contributions

ES, VC, UB, and MM conceived and designed the experiments. ES performed the experiments. MM, UB, and FP followed the patients, including planning clinical visits and blood sample collection, follow-up and clinical data collection. PDB performed statistical analysis. ES, IC, PDB, VC, MM, and VZ analyzed the results. ES, MM, UB, and VC wrote and revised the paper. All authors reviewed the manuscript.

## Conflict of Interest

The authors declare that the research was conducted in the absence of any commercial or financial relationships that could be construed as a potential conflict of interest.
