# Supplementary material for: Prognostic Stratification of Metastatic Prostate Cancer Patients Treated With Abiraterone and Enzalutamide Through an Integrated Analysis of Circulating Free microRNAs and Clinical Parameters
Source: Front Oncol. 2021 Mar 16;11:626104. doi: 10.3389/fonc.2021.626104 (PMC8009625; doi:10.3389/fonc.2021.626104)
Supplement: Supplementary file 1 [file Data_Sheet_1.PDF]

**SUPPLEMENTAL INFORMATION**

**Table S1**. Characteristics of patients in the study.

| **Patient #** | **Treatment in study** | **Haemolysis*** | **miR-1228** Ct | **miR-141/1228** $2^{-\Delta Ct}$ | **miR-21/1228** $2^{-\Delta Ct}$ | **miR-223/1228** $2^{-\Delta Ct}$ | **tCRPC** (months) | **Hb** (g/L) | **PFS** (months) | **OS** (months) | **Previous therapy** | **Post-progression therapy** |
|---|---|---|---|---|---|---|---|---|---|---|---|---|
| **#1** | Abiraterone Acetate | 0.047 | 28.21 | 0.0038 | 2.2736 | 4.2428 | 49.7 | 140 | 3.73 | 37.07 | 0 | Docetaxel > Cabazitaxel |
| **#2** | Enzalutamide | 0.028 | 27.82 | 0.0057 | 3.6427 | 4.0139 | 19.2 | 131 | 39.40 | 42.17 | 0 | Abiraterone |
| **#3** | Abiraterone Acetate | 0.024 | 28.85 | 0.0196 | 2.1214 | 11.5115 | 32.3 | 148 | 17.23 | 32.77 | 0 | Docetaxel |
| **#4** | Enzalutamide | 0.051 | 29.25 | 0.0403 | 11.3924 | 15.5625 | 45.0 | 139 | 35.70 | 40.87 | 0 | Docetaxel > $Radium^{223}$ |
| **#5** | Enzalutamide | 0.041 | 27.76 | 0.0121 | 3.1821 | 2.4538 | 48.9 | 135 | 15.17 | 40.63 | 0 | Docetaxel >Cabazitaxel >Mitoxantrone |
| **#6** | Enzalutamide | 0.065 | 27.17 | 0.0278 | 3.7711 | 4.8906 | 35.0 | 130 | 30.03 | 38.47 | 0 | $Radium^{223}$ |
| **#7** | Abiraterone Acetate | 0.021 | 27.81 | 0.0277 | 6.5887 | 13.5479 | 33.1 | 135 | 39.90 | 39.90 | Docetaxel | N/A |
| **#8** | Enzalutamide | 0.014 | 29.09 | 0.0135 | 10.2319 | 5.4076 | 19.0 | 112 | 31.37 | 36.57 | 0 | Ciclofosfamide |
| **#9** | Enzalutamide | 0.028 | 27.15 | 0.0065 | 4.6589 | 5.1694 | 30.4 | 140 | 24.80 | 39.87 | 0 | No therapy |
| **#10** | Enzalutamide | 0.051 | 27.40 | 0.0139 | 5.7958 | 4.3469 | 111.3 | 122 | 6.80 | 6.80 | 0 | No therapy |
| **#11** | Enzalutamide | 0.125 | 27.89 | 0.0170 | 13.1775 | 12.1257 | 79.4 | 168 | 20.40 | 33.70 | Docetaxel | No therapy |
| **#12** | Enzalutamide | 0.056 | 23.53 | 0.2457 | 1.4743 | 0.8706 | 11.8 | 132 | 2.10 | 8.53 | 0 | $Radium^{223}$ |
| **#13** | Enzalutamide | 0.045 | 27.68 | 0.2661 | 18.0009 | 12.2525 | 13.1 | 129 | 6.97 | 37.33 | 0 | Docetaxel > Abiraterone > Cabazitaxel |
| **#14** | Enzalutamide | 0.038 | 26.05 | 0.0711 | 7.8354 | 6.3864 | 56.4 | 135 | 38.50 | 38.50 | 0 | No therapy |
| **#15** | Abiraterone Acetate | 0.024 | 27.57 | 0.0375 | 9.2856 | 12.5969 | 4.2 | 135 | 30.33 | 37.10 | 0 | Docetaxel > Cabazitaxel |
| **#16** | Abiraterone Acetate | 0.053 | 25.87 | 0.0123 | 4.3772 | 10.9663 | 15.2 | 135 | 11.83 | 12.53 | 0 | No therapy |
| **#17** | Enzalutamide | 0.025 | 27.14 | 0.0235 | 2.9794 | 11.5915 | 78.0 | 133 | 36.70 | 36.70 | 0 | N/A |
| **#18** | Enzalutamide | 0 | 27.06 | 0.0374 | 11.7127 | 13.0412 | 47.8 | 100 | 4.13 | 4.13 | 0 | No therapy |

| | | | | | | | | | | | | |
|---|---|---|---|---|---|---|---|---|---|---|---|---|
| **#19** | Abiraterone Acetate | 0.055 | 24.44 | 0.2956 | 2.0023 | 0.7178 | 9.1 | 113 | 5.80 | 5.93 | 0 | No therapy |
| **#20** | Enzalutamide | 0.005 | 24.64 | 0.0252 | 0.9255 | 1.0163 | 8.0 | 110 | 2.10 | 3.43 | 0 | No therapy |
| **#21** | Enzalutamide | 0.068 | 27.29 | 0.0171 | 6.4086 | 10.7406 | 46.3 | 131 | 12.60 | 21.20 | 0 | $Radium^{223}$ |
| **#22** | Abiraterone Acetate | 0.058 | 26.74 | 0.0224 | 4.5473 | 5.5983 | 60.8 | 114 | 19.57 | 35.93 | 0 | Docetaxel > Enzalutamide > Cabazitaxel |
| **#23** | Enzalutamide | 0.002 | 23.61 | 0.2293 | 1.8088 | 0.3143 | 38.1 | 119 | 12.17 | 17.53 | 0 | No therapy |
| **#24** | Abiraterone Acetate | 0.09 | 27.27 | 0.0108 | 6.2767 | 10.0561 | 87.2 | 134 | 29.40 | 33.60 | 0 | Docetaxel > Cabazitaxel |
| **#25** | Abiraterone Acetate | 0.1 | 25.91 | 0.2003 | 5.3147 | 6.7039 | 39.8 | 133 | 28.67 | 33.57 | Docetaxel | Cabazitaxel |
| **#26** | Enzalutamide | 0.076 | 26.13 | 0.0077 | 8.9074 | 20.3930 | 66.6 | 131 | 17.23 | 32.43 | Docetaxel | Cabazitaxel |
| **#27** | Enzalutamide | 0.083 | 25.23 | 0.0046 | 3.7581 | 6.5432 | 28.9 | 139 | 31.80 | 32.50 | 0 | Docetaxel |
| **#28** | Enzalutamide | 0.051 | 26.33 | 0.0052 | 4.7404 | 11.4320 | 20.7 | 142 | 32.40 | 32.40 | 0 | best supportive care |
| **#29** | Enzalutamide | 0.012 | 26.35 | 0.0409 | 2.6851 | 4.1125 | 83.0 | 118 | 9.60 | 20.70 | 0 | best supportive care |
| **#30** | Abiraterone Acetate | 0.076 | 27.49 | 0.0409 | 9.3827 | 10.4831 | 46.6 | 128 | 30.50 | 30.50 | Docetaxel | N/A |
| **#31** | Enzalutamide | 0.024 | 26.83 | 0.3078 | 5.7557 | 4.0558 | 6.6 | 127 | 8.80 | 20.33 | 0 | Docetaxel |

*Haemolysis was measured as free Hb (g/L) corrected for lipoprotein content as described in the Materials and Methods.

**Table S2**. Association of patients' clinical and biological characteristics with PFS.

| | | Univariate analysis | | | | | | Multivariate analysis | | |
|---|---|---|---|---|---|---|---|---|---|---|
| **Variable** | | **E/N** | **Median (95% CI)** | ***Logrank p-value$^{BH}$*** | **HR** | **95% CI** | **p-value$^{BH}$** | **HR** | **95% CI** | **p-value** |
| **PFS** | | 26/31 | 19.3 (11.7-29.6) | | | | | | | |
| **Treatment** | Abiraterone | 8/10 | 23.8 (3.7-29.9) | 0.8412 | 1 | | | | | |
| | Enzalutamide | 18/21 | 17.0 (8.7-30.9) | | 1.09 | 0.47-2.53 | 0.8405 | | | |
| **PSA (ng/mL)** | ≤7.45 | 6/8 | 32.5 (4.1-) | 0.2618 | 1 | | | | | |
| | >7.45 | 20/23 | 14.9 (8.7-28.3) | | 1.83 | 0.73-4.60 | 0.2650 | | | |
| **tCRPC (months)** | ≤15.2 | 7/7 | 6.9 (2.1-11.7) | **0.0039** | 4.09 | 1.62-10.33 | **0.0068** | 4.8 | 1.65-14 | **0.004** |
| | >15.2 | 19/24 | 26.3 (14.9-30.9) | | 1 | | | | | |
| **N/L** | ≤1.48 | 8/10 | 29.4 (12.0-31.3) | 0.2618 | 1 | | | | | |
| | >1.48 | 18/21 | 12.4 (6.7-28.3) | | 1.71 | 0.74-3.94 | 0.2650 | | | |
| **Hb (g/L)** | ≤127 | 9/9 | 8.7 (2.1-19.3) | **0.0039** | 3.67 | 1.54-8.75 | **0.0068** | 2.3 | 0.70-7.2 | 0.171 |
| | >127 | 17/22 | 28.6 (14.9-35.2) | | 1 | | | | | |
| **ECOG PS** | 0 | 7/10 | 28.6 (8.7-) | 0.2618 | 1 | | | | | |
| | 1-2 | 19/21 | 17.0 (6.7-29.6) | | 1.74 | 0.73-4.16 | 0.2650 | | | |
| **Gleason score** | ≤7 | 5/7 | 24.4 (3.7-) | 0.8221 | 1 | | | | | |
| | >7 | 19/21 | 19.3 (9.5-29.9) | | 1.18 | 0.43-3.22 | 0.8255 | | | |
| **miR-21/1228** | ≤2.69 | 7/7 | 5.7 (2.1-) | **0.0002** | 7.38 | 2.56-21.25 | **0.0021** | 4.8 | 1.29-17.8 | **0.019** |
| | >2.69 | 19/24 | 28.6 (19.3-35.2) | | 1 | | | | | |
| **miR-141/1228** | ≤0.20 | 21/26 | 26.3 (14.9-30.9) | **0.0010** | 1 | | | | | |
| | >0.20 | 5/5 | 6.9 (2.1-) | | 7.43 | 2.18-25.37 | **0.0068** | 1.2 | 0.27-5.6 | 0.79 |
| **miR-223/1228** | ≤4.35 | 10/10 | 7.7 (2.1-12.0) | **0.0039** | 3.52 | 1.52-8.13 | **0.0068** | 2.0 | 0.62-6.3 | 0.246 |
| | >4.35 | 16/21 | 29.0 (17.0;31.3) | | 1 | | | | | |

Abbreviations: PSA, prostate-specific antigen; tCRPC, time to development of castration-resistance; N, neutrophils; Ly, lymphocytes; N/L, neutrophil-lymphocyte ratio; Hb, hemoglobin; ECOG PS, Eastern Cooperative Oncology Group Performance Status; BH, Benjamini-Hochberg correction.

**Table S3**. Association of patients' clinical and biological characteristics with OS.

| | | Univariate analysis | | | | | | Multivariate analysis | | |
|---|---|---|---|---|---|---|---|---|---|---|
| | | **E/N** | **Median (95% CI)** | ***Logrank p-value$^{BH}$*** | **HR** | **95% CI** | **p-value$^{BH}$** | **HR** | **95% CI** | **p-value** |
| **OS** | | 13/31 | - | | | | | | | |
| **Treatment** | Abiraterone | 3/10 | - | 0.4376 | 1 | | | | | |
| | Enzalutamide | 10/21 | 36.0 (20.0-) | | 1.66 | 0.46-6.05 | 0.4424 | | | |
| **PSA (ng/mL)** | ≤7.45 | 1/8 | - | 0.1691 | 1 | | | | | |
| | >7.45 | 12/23 | 36.0 (20.0-) | | 4.45 | 0.58-34.27 | 0.2171 | | | |
| **tCRPC (months)** | ≤15.2 | 5/7 | 12.4 (3.4-) | **0.0405** | 3.5 | 1.14-10.94 | **0.0594** | 7.61 | 1.43-40.5 | **0.017** |
| | >15.2 | 8/24 | - | | 1 | | | | | |
| **N/L** | ≤1.48 | 3/10 | - | 0.2949 | 1 | | | | | |
| | >1.48 | 10/21 | - | | 2.05 | 0.56-7.47 | 0.3067 | | | |
| **Hb (g/L)** | ≤127 | 8/9 | 17.3 (3.4-) | **0.0003** | 8.01 | 2.55-25.16 | **0.0036** | 8.97 | 2.24-35.8 | **0.002** |
| | >127 | 5/22 | - | | 1 | | | | | |
| **ECOG PS** | 0 | 2/10 | - | 0.1691 | 1 | | | | | |
| | 1-2 | 11/21 | 33.2 (12.4-) | | 3.12 | 0.69-14.11 | 0.2171 | | | |
| **Gleason score** | ≤7 | 1/7 | - | 0.1808 | 1 | | | | | |
| | >7 | 11/21 | 36.0 (17.3-) | | 4.10 | 0.53-31.91 | 0.2225 | | | |
| **miR-21/1228** | ≤2.69 | 6/7 | 17.3 (36.0-) | **0.0067** | 5.16 | 1.70-15.70 | **0.0191** | 5.77 | 1.01-33.1 | **0.049** |
| | >2.69 | 7/24 | - | | 1 | | | | | |
| **miR-141/1228** | ≤0.20 | 9/26 | - | **0.0405** | 1 | | | | | |
| | >0.20 | 4/5 | 17.3 (5.8-) | | 3.82 | 1.14-12.79 | **0.0594** | 1.87 | 0.32-10.8 | 0.484 |
| **miR-223/1228** | ≤4.35 | 7/10 | 18.7 (3.4-) | **0.0357** | 3.82 | 1.27-11.52 | **0.0577** | 0.84 | 0.12-5.9 | 0.862 |
| | >4.35 | 6/21 | - | | 1 | | | | | |

Abbreviations: PSA, prostate-specific antigen; tCRPC, time to development of castration-resistance; N, neutrophils; Ly, lymphocytes; N/L, neutrophil-lymphocyte ratio; Hb, hemoglobin; ECOG PS, Eastern Cooperative Oncology Group Performance Status. BH: Benjamini-Hochberg correction.

**Table S4**. Correlations among patients' clinical and biological characteristics.

| Row | Column | Cor (Spearman) | *p-value*[BH] |
|---|---|---|---|
| PSA | tCRPC | -0.14 | *0.8346* |
| PSA | N/L | -0.02 | *0.9074* |
| tCRPC | N/L | -0.09 | *0.8964* |
| PSA | Hb | 0.07 | *0.8964* |
| tCRPC | Hb | 0.07 | *0.8964* |
| N/L | Hb | -0.07 | *0.8964* |
| PSA | miR-21/1228 | -0.26 | *0.5586* |
| tCRPC | miR-21/1228 | 0.17 | *0.7720* |
| N/L | miR-21/1228 | -0.26 | *0.5586* |
| Hb | miR-21/1228 | 0.06 | *0.7558* |
| PSA | miR-141/1228 | 0.21 | *0.2567* |
| tCRPC | miR-141/1228 | -0.24 | *0.5655* |
| N/L | miR-141/1228 | -0.02 | *0.9074* |
| Hb | miR-141/1228 | -0.45 | ***0.1218*** |
| miR-21/1228 | miR-141/1228 | 0.05 | *0.8964* |
| PSA | miR-223/1228 | -0.29 | *0.5586* |
| tCRPC | miR-223/1228 | 0.19 | *0.7243* |
| N/L | miR-223/1228 | -0.04 | *0.8964* |
| Hb | miR-223/1228 | 0.37 | ***0.2821*** |
| miR-21/1228 | miR-223/1228 | 0.71 | ***0*** |
| miR-141/1228 | miR-223/1228 | -0.13 | *0.8774* |

Abbreviations: PSA, prostate-specific antigen; tCRPC, time to development of castration-resistance; N, neutrophils; Ly, lymphocytes; N/L, neutrophil-lymphocyte ratio; Hb, hemoglobin; ECOG PS, Eastern Cooperative Oncology Group Performance Status; BH, Benjamini-Hochberg correction.

**Figure S1**. Kaplan-Maier curves showing clinical and biological characteristics identified as significant in univariate analysis for PFS (left panels) and OS (right panels).

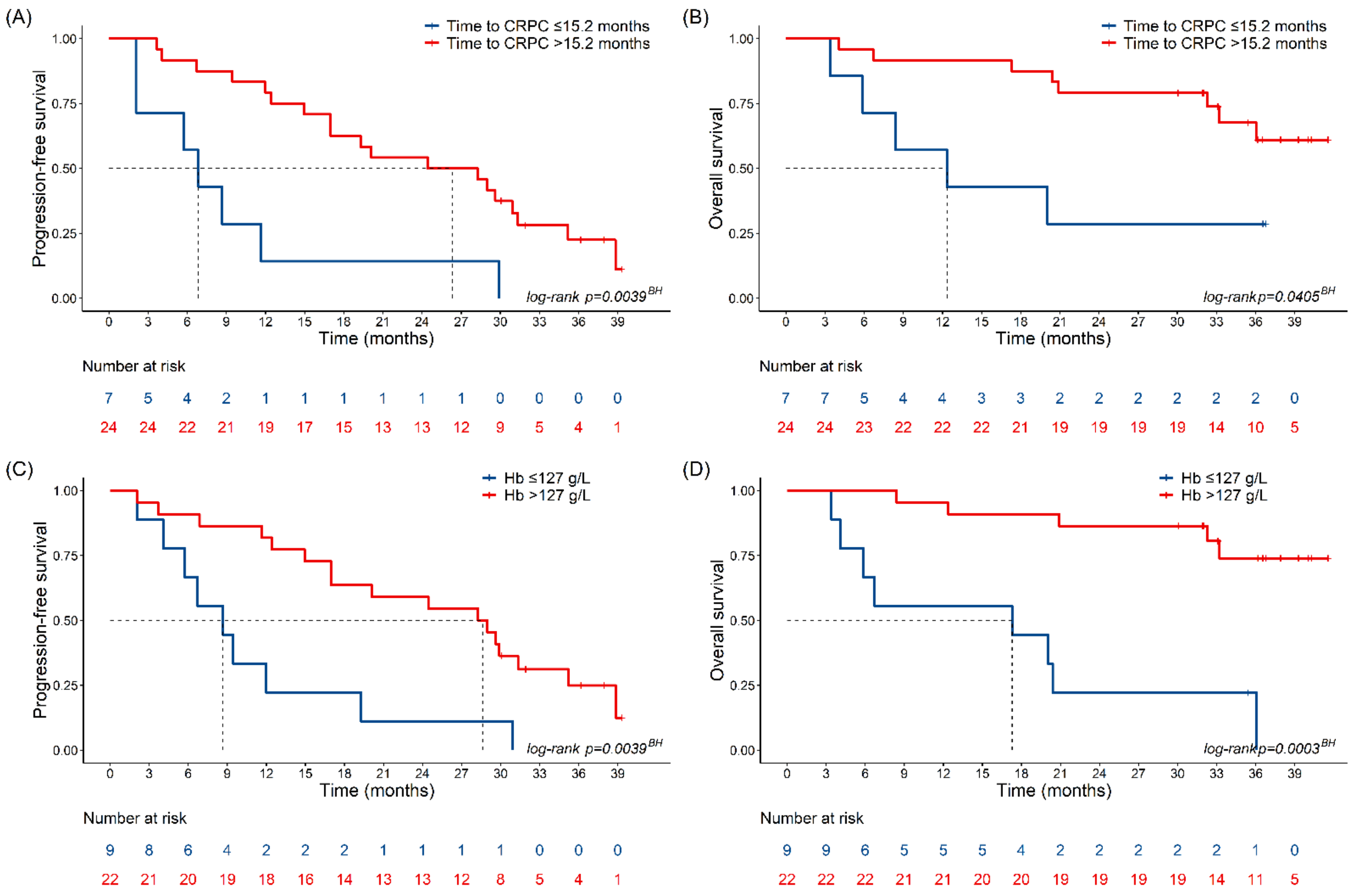

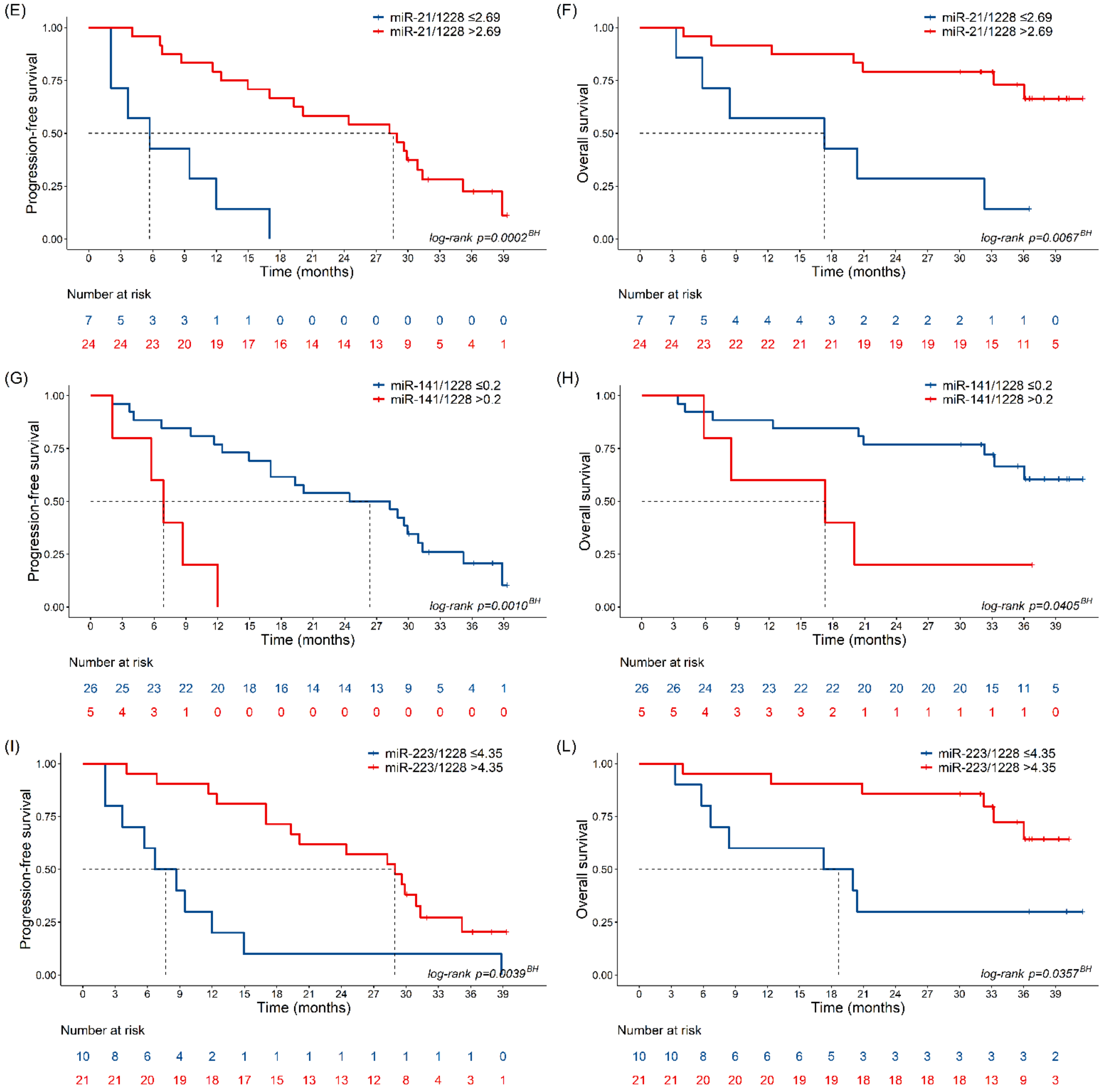
(E)
miR-21/1228 ≤2.69
miR-21/1228 >2.69
Progression-free survival
Time (months)
log-rank p=0.0002^BH
Number at risk
7 5 3 3 1 1 0 0 0 0 0 0 0 0
24 24 23 20 19 17 16 14 14 13 9 5 4 1
(F)
miR-21/1228 ≤2.69
miR-21/1228 >2.69
Overall survival
Time (months)
log-rank p=0.0067^BH
Number at risk
7 7 5 4 4 4 3 2 2 2 2 1 1 0
24 24 23 22 22 21 21 19 19 19 19 15 11 5
(G)
miR-141/1228 ≤0.2
miR-141/1228 >0.2
Progression-free survival
Time (months)
log-rank p=0.0010^BH
Number at risk
26 25 23 22 20 18 16 14 14 13 9 5 4 1
5 4 3 1 0 0 0 0 0 0 0 0 0 0
(H)
miR-141/1228 ≤0.2
miR-141/1228 >0.2
Overall survival
Time (months)
log-rank p=0.0405^BH
Number at risk
26 26 24 23 23 22 22 20 20 20 20 15 11 5
5 5 4 3 3 3 2 1 1 1 1 1 1 0
(I)
miR-223/1228 ≤4.35
miR-223/1228 >4.35
Progression-free survival
Time (months)
log-rank p=0.0039^BH
Number at risk
10 8 6 4 2 1 1 1 1 1 1 1 1 0
21 21 20 19 18 17 15 13 13 12 8 4 3 1
(L)
miR-223/1228 ≤4.35
miR-223/1228 >4.35
Overall survival
Time (months)
log-rank p=0.0357^BH
Number at risk
10 10 8 6 6 6 5 3 3 3 3 3 3 2
21 21 20 20 20 19 19 18 18 18 18 13 9 3
